# Nebulised dornase alfa versus placebo or hypertonic saline in adult critically ill patients: a systematic review of randomised clinical trials with meta-analysis and trial sequential analysis

**DOI:** 10.1186/s13643-015-0142-z

**Published:** 2015-11-08

**Authors:** Casper Claudius, Anders Perner, Morten Hylander Møller

**Affiliations:** Department of Intensive Care 4131, Copenhagen University Hospital Rigshospitalet, Blegdamsvej 9, 2100 Copenhagen, Denmark

**Keywords:** Systematic review, Meta-analysis, Trial sequential analysis, Dornase alfa, Pulmozyme, Critically ill patients

## Abstract

**Background:**

Nebulised dornase alfa is used off-label in critically ill patients. We aimed to assess the benefits and harms of nebulised dornase alfa versus placebo, no prophylaxis, or hypertonic saline on patient-important outcome measures in adult critically ill patients.

**Methods:**

We performed a systematic review with meta-analysis and trial sequential analysis (TSA) using the Cochrane Collaboration methodology. Eligible trials were randomised clinical trials comparing nebulised dornase alfa with placebo, no prophylaxis, or hypertonic saline. The predefined outcome measures were all-cause mortality, duration of mechanical ventilation, length of stay, and adverse events. Two reviewers independently assessed trials for inclusion, data extraction, and risk of bias. Risk ratios (RRs) with 95 % confidence intervals (CIs) were estimated by conventional cumulative meta-analysis, and the robustness of the primary estimate was assessed by TSA.

**Results:**

Two trials (*n* = 63) were included; both were judged to have high risk of bias. There was no statistically significant difference in mortality (random effects model RR (95 % CI) 0.73 (0.09–5.77); *P* = 0.24; *I*^2^ = 30 %). TSA could not be conducted because less than 1 % of the required information size had been accrued. None of the two trials reported adequate and detailed data on any of the secondary outcome measures.

**Conclusions:**

We found very low quantity and quality of evidence for use of nebulised dornase alfa in adult critically ill patients in this systematic review with meta-analysis.

**Systematic review registration:**

The International Prospective Register of Systematic Reviews (PROSPERO), no. CRD442015016047.

**Electronic supplementary material:**

The online version of this article (doi:10.1186/s13643-015-0142-z) contains supplementary material, which is available to authorized users.

## Background

Dornase alfa (Pulmozyme) is a nebulised recombinant human deoxyribonuclease I (rhDNase) approved and used in patients with cystic fibrosis (CF) [1]. It is a 260-amino acid glycoprotein with 2.5 mg of active drug for nebulization once or twice daily. It reduces the viscosity of the mucus in a dose-dependent fashion in patients with CF, and long-term use results in improved lung function [2], but the effect on mortality is inconclusive [2].

There appears to be an increasing use of dornase alfa outside the clinical context of CF, including in patients with empyema [3], atelectasis [4], asthma [5], and sinusitis [6]. A possible benefit of dornase alfa in adult critically ill patients with atelectasis and mucus plugging has been suggested, and clinical use in the intensive care unit (ICU) has been reported [1]. However, in a recent review, it was concluded that there is insufficient evidence for the efficacy of dornase alfa use in pediatric patients with non-CF pulmonary atelectasis [7].

The aim of the present systematic review was to assess the benefits and harms of dornase alfa versus placebo or hypertonic saline on patient-important outcome measures in adult critically ill patients. We hypothesized that there is very little evidence supporting off-label use of dornase alfa in adult critically ill patients.

## Methods

This systematic review is based on the methodology recommended by the Cochrane Collaboration [8], and the manuscript has been prepared according to the PRISMA statement (Additional file 1) [9]. The protocol has been published in the International Prospective Register of Systematic Reviews (PROSPERO), no. CRD42015016047.

### Eligibility criteria

Potentially eligible trials had to be randomised; include adult critically ill patients; have an intervention group that received inhaled dornase alfa in any dose and of any duration; and a control group that received no treatment, placebo, or hypertonic saline. We included trials irrespective of language, blinding, publication status, and number of intervention groups. Exclusion criteria were trials in animals, trials in pediatric patients, trials in patients with CF, and trials not reporting the patient-important outcome measures [10, 11] outlined in the protocol, e.g., changes on chest radiograph or in gas exchange.

### Outcome measures

The predefined primary and secondary outcome measures were as follows: all-cause mortality at the longest follow-up (primary), duration of mechanical ventilation, length of stay in ICU, length of stay in hospital, and adverse events (secondary). The outcome measures were defined as by the authors of the included trials.

### Search strategy

We framed the following clinical research question: “Is treatment with nebulised dornase alfa in adult critically ill patients superior to no treatment, placebo or hypertonic saline?”

A population, intervention, comparator, and outcomes (PICO)-based question and literature search was created [12]:

- *Population*: critically ill OR icu OR intensive care unit OR intensive care
- *Intervention*: recombinant human deoxyribonuclease OR dornase alpha OR recombinant human DNase OR rhDNase OR Pulmozyme
- *Comparator*: control OR placebo OR hypertonic saline
- *Outcomes*: mortality OR death OR mechanical ventilation OR intermittent positive pressure ventilation OR length of stay OR LOS OR adverse events OR morbidity

The following databases were searched for literature: MEDLINE including MeSH (January 1966 to January 2015), Embase (January 1980 to January 2015) and the Cochrane Library (Issue 3, January 2015). The detailed search strategy is available as an additional file (Additional file 2). We also hand-searched the reference lists of the included trials and other relevant reviews, and data from unpublished trials were explored. The electronic literature search was last updated 9 September 2015.

### Study selection

Two authors (CC and MHM) independently reviewed all titles and abstracts identified in the literature search and excluded trials that were obviously not relevant. The remaining trials were evaluated in full-text. Disagreements were resolved with AP.

### Data extraction

Two authors (CC and MHM) independently extracted information from each included trial using a data extraction form. The extracted information included trial characteristics (year of publication, trial duration, and country); characteristics of the trial participants (inclusion and exclusion criteria); type of intervention/control (name, dosing, duration, and comparator); outcomes; and risk of bias. The corresponding authors of the included trials were sought contacted electronically for additional details; however, no one responded.

### Risk of bias assessment

In order to evaluate the risk of systematic errors in the included trials, two authors (CC and MHM) independently assessed the risk of bias as advised by the Cochrane Collaboration [8], including the domains of random sequence generation, allocation concealment, blinding, incomplete outcome data, selective outcome reporting, baseline imbalance, and bias due to vested financial interest. If one or more domains were judged as being high or unclear, we classified the trial as having an overall high risk of bias [8]. Disagreements were resolved with AP.

### Statistical analyses

For each included trial, we calculated relative risk (RR) with 95 % confidence intervals (CIs) for dichotomous outcome measures and mean difference (MD) with 95 % CI for continuous outcome measures, and we pooled these measures in conventional cumulative meta-analyses. Statistical heterogeneity among trials was quantified with inconsistency factor (*I*^2^) [13] and diversity (*D*^2^) statistics [14]. If the *I*^2^ statistic was 0, we reported the results from a fixed effects model, and if the *I*^2^ statistic was >0, we reported results from a random effects model (the most conservative estimate).

We used chi-squared test to provide an indication of heterogeneity between studies (test-of-interaction); *P* < 0.10 was considered significant.

The risk of random errors in the cumulative meta-analyses was assessed by trial sequential analyses (TSA) [14–16]. TSA is a sample size calculation (interim analysis) for meta-analyses that widens the confidence intervals in case data are too sparse to draw firm conclusions (repetitive testing). We conducted TSA with the intention to maintain an overall 5 % risk of a type I error and a power of 80 %. For the calculation of the required information size, we used a 20 % relative risk reduction (RRR) of the intervention effect for the dichotomous outcome measures, and for the continuous outcome measures, the required information size was based on a calculated value based on the included trials [17]. If—according to the TSA—less than 5 % of the *D*^2^-adjusted required information size (DIS) has been accrued, no TSA details or plots are presented.

A sensitivity analysis with application of continuity correction in trials of zero events was conducted [18].

Risk of small trial bias by means of funnel plot was abandoned because less than ten trials were included [8].

Review Manager 5.2 (the Nordic Cochrane Centre, The Cochrane Collaboration, Copenhagen, Denmark) was used for the conventional meta-analyses, and for the TSA, we used the TSA program version 0.9 beta [17].

### Subgroup analyses

The following predefined subgroup analyses were planned:

1. Comparing estimates of the pooled intervention effect in trials with low risk of bias to estimates from trials with high risk of bias (hypothesized direction of subgroup effect: increased intervention effect in trials with high risk of bias).
2. Comparing estimates of the pooled intervention effect in trials conducted in mechanically ventilated patients versus non-mechanically ventilated patients (hypothesized direction of subgroup effect: increased intervention effect in mechanically ventilated patients).
3. Comparing estimates of the pooled intervention effect in trials using no treatment/placebo as comparator versus trials using hypertonic saline as comparator (hypothesized direction of subgroup effect: increased intervention effect in trials using placebo as comparator).

Only the third subgroup analysis could be conducted and reported, as neither trial had overall low risk of bias nor included non-mechanically ventilated patients.

## Results

A total of two trials were included [19, 20] (Additional file 1 and Table 1). The main reason for exclusion was that the trials were conducted in patients with CF and/or pediatric patients. One trial was excluded because the population was non-critically ill patients with asthma (treatment in the emergency department with only half of the patients subsequently hospitalized) [5].

**Table 1:** Characteristics of the included trials

Trial	Number	Setting	Trial duration	ICU	Population		Intervention	Comparator	Outcomes
					Inclusion criteria	Exclusion criteria			
Youness et al. [20]	33	Single-centre US	24 months	Mixed	Intubated mechanically ventilated patients with new-onset (<48 h) atelectasis	CF, uncontrolled asthma, COPD, pneumothorax, pleura effusion, tumor-associated atelectasis, severe hypoxemia, hemodynamic instability, allergy to dornase alfa, use of nebulized acetylcysteine and pregnancy	Nebulized dornase alfa 2.5 mg twice daily in 7 days or until resolution of atelectasis	(1) Placebo (isotonic saline) same volume, dosage, and duration of treatment	Mortality
								(2) Hypertonic saline (same volume, dosage, and duration of treatment)	
Zitter et al. [19]	30	Single-centre US	12 months	Mixed	Mechanically ventilated patients aged >18 years with new-onset (<24 h) atelectasis	Quadriplegia, chronic ventilator dependence, pneumothorax, frank hemoptysis, elevated intracranial pressure, pregnancy or active nursing, concurrent use of other investigational drugs, and allergy to dornase alfa, Chinese hamster ovary-derived biologics or other components of the active component	Nebulized dornase alfa 2.5 mg twice daily until extubation, death or transfer (maximum 30 days)	Placebo (isotonic saline) same volume, dosage, and duration of treatment	Mortality

*CF* Cystic fibrosis, *COPD* chronic obstructive pulmonary disease

### Characteristics of trials

Both trials were single-centre trials from the USA and included patients from mixed ICUs (Table 1).

### Participants

The two included trials enrolled a total of 63 adult critically ill patients, whom were all mechanically ventilated. The trial inclusion and exclusion criteria were somewhat homogenous (Table 1).

### Intervention and comparators

Both trials evaluated nebulised dornase alfa 2.5 mg twice daily versus nebulised placebo twice daily, and one [20] also assessed nebulised hypertonic saline twice daily as comparator (three-armed design).

### Risk of bias

**Fig. 1:**
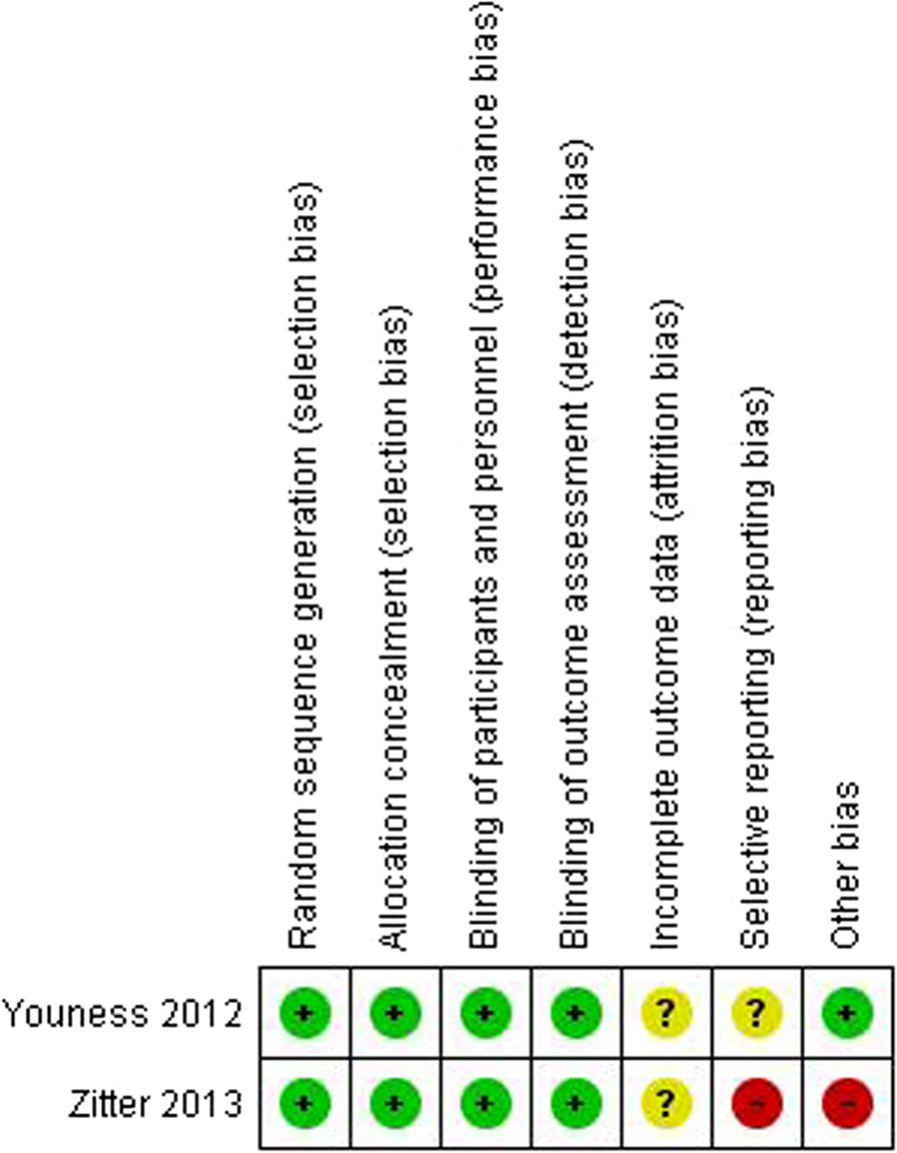
Risk of bias summary. Review of authors’ judgements about each risk of bias item for each included study. *Red* = high risk; *green* = low risk; *yellow* = unclear

No trials were judged to be of low risk of bias in all six domains (Fig. 1). The main reasons for high risk of bias were unclear/high risk of attrition bias and reporting bias, as secondary outcome measures were inadequately reported or supplied upon request, and because of inadequate duration of follow-up [19]. Furthermore, one trial had potential financial bias because the company providing dornase alfa funded the trial [19].

### Outcome measures

#### All-cause mortality

**Fig. 2:**
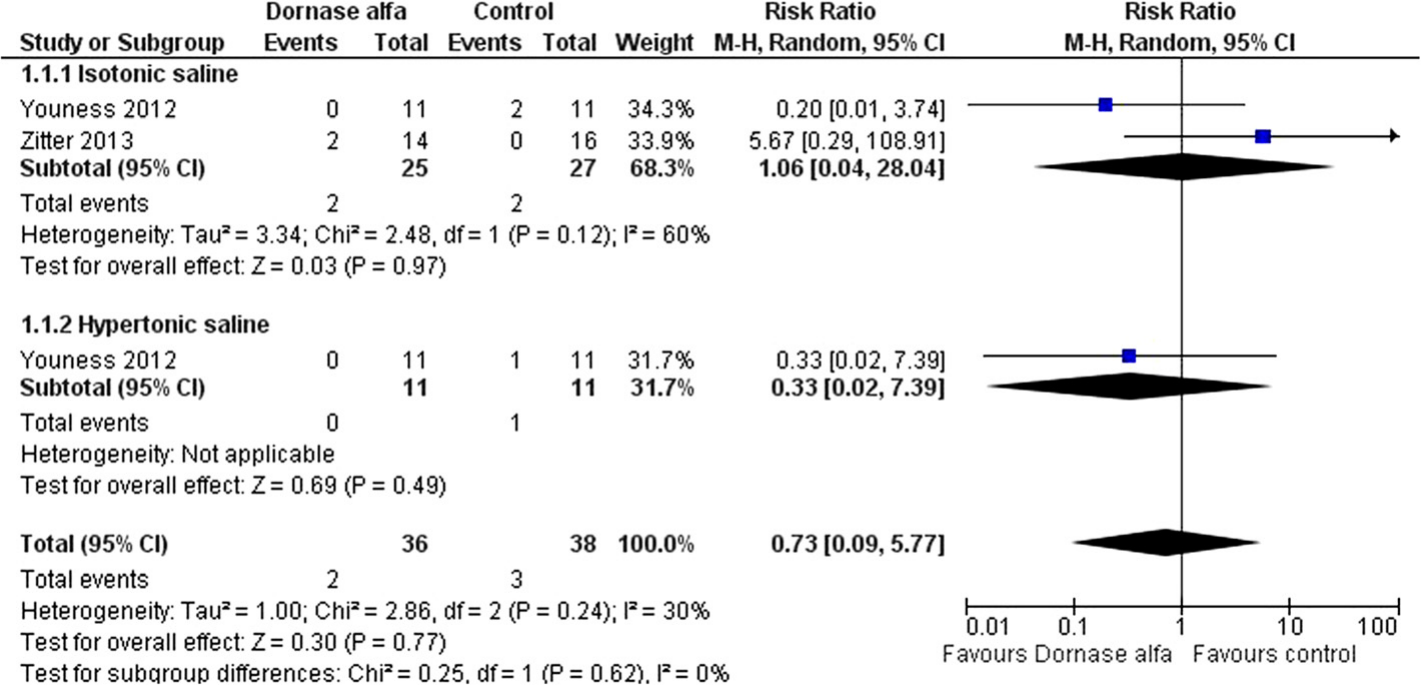
Dornase alfa and all-cause mortality. Size of squares for risk ratio reflects the weight of trial in pooled analyses. *Horizontal bars* represent 95 % confidence intervals

Mortality data were obtained from both trials including 63 patients in total. The conventional meta-analysis showed no difference in mortality in patients treated with dornase alfa compared with the control group: random effects model RR (95 % CI) 0.73 (0.09–5.77); *P* = 0.24; *I*^2^ = 30 % (Fig. 2). The subgroup analysis of trials using placebo as comparator versus trials using hypertonic saline as comparator showed no statistically significantly increased intervention effect in trials using placebo (test of interaction *P* = 0.62). TSA could not be conducted due to too few data (DIS <1 %). The sensitivity analysis with continuity correction of the no-event trials was consistent with the primary summary estimate.

#### Secondary outcome measures

Both trials reported that no adverse events were observed; however, no detailed information on this issue were available. No trials reported adequate data on other secondary outcome measures, and the corresponding authors did not supply these data upon request.

## Discussion

In the present systematic review of RCTs, we found very low level of evidence for benefit or harm of dornase alfa use in adult critically ill patients in terms of patient- important outcome measures.

### Mortality

The conventional cumulative meta-analysis of mortality showed no statistically significant benefit or harm of dornase alfa. No subgroup difference was present between placebo and hypertonic saline. Importantly, TSA highlighted the considerable lack of data accrued, as less than 1 % of the required information size had been accrued. Finally, both included trials had high risk of bias, which could result in inflated point estimates. Considering the high risk of bias and the very limited data, no firm evidence for benefit or harm of dornase alfa use in adult critically ill patients in terms of mortality exists [21, 22].

### Secondary outcome measures

The predefined patient-important secondary outcome measures [10, 11], including duration of mechanical ventilation, adverse events, and length of stay, were inadequately reported in the included trials and were not supplied upon request to the authors. Accordingly, the effect of dornase alfa on patient-important outcome measures in adult critically ill patients is virtually unknown.

### Strengths and limitations of the review

The compliance with the recommendations of the Cochrane Collaboration is a strength of the present systematic review, including a pre-experimentally published protocol, a systematic literature search with no language restrictions, independent literature search, data extraction and risk of bias assessment by two authors, contact to authors for further details, and the inclusion of trials irrespective of publication status. In addition, we evaluated the risk of random errors with the application of TSA to increase the robustness of the analyses. We excluded trials not reporting patient important- outcome measures in order to make the results relevant for patients and clinical practice [10, 11]. We did not define the outcome measures evaluated; rather, we used the definitions proposed by the authors, which may have resulted in some degree of trial heterogeneity. Finally, results of the predefined subgroup analyses should be interpreted critically, as very few trials were included in the primary analyses.

### Relation to other reviews and implications for future research

No previous systematic reviews on the use of dornase alfa in adult critically ill patients have been published. As highlighted in the present review, there is a lack of firm evidence for the use of dornase alfa in this population, as existing data are very limited (two RCTs, *n* = 63) and of low quality.

In 2014, Thornby and colleagues summarized existing evidence on dornase alfa use in pediatric patients with non-CF pulmonary atelectasis [7]. A total of eight trials (one RCT) and 12 case-series were included. The overall risk of bias was high, and trials suffered from significant heterogeneity in terms of the population of interest, the intervention, the comparator, and the outcomes of interest. Consequently, the authors concluded that there is insufficient evidence for the efficacy of dornase alfa in the treatment of atelectasis in pediatric patients. However, the authors suggested that dornase alfa may be useful as a second-line treatment option if conventional non-pharmacological treatment for atelectasis fails. We believe this statement can be challenged. In everyday clinical practice, it is essential to balance the potential benefits and harms of an intervention. In patients with CF, there is firm evidence that long-term treatment with dornase alfa improves lung function; however, the effect on mortality is unknown [2]. Importantly, there seems to be an increased risk of adverse events when using dornase alfa as compared to placebo, including rash and voice alterations [2]. In recent years, a number of interventions used in the ICU have proved harmful following adequate evaluation in high-quality trials [23–27]. Accordingly, short-term use of dornase alfa in critically ill patients outside the clinical context of CF may be inappropriate without firm evidence for patient-centered benefit and no (low) risk of adverse events. Moreover, when two interventions are (presumed) equivalent, it is of clinical and financial interest to assess costs. In 2001, Grieve and colleagues performed a cost-effectiveness analysis of dornase alfa use in children with CF [28]. The drug cost per day was increased by a factor of 50 (£0.38 vs. £20.39) by using dornase alfa as compared to hypertonic saline. Accordingly, it is likely that the use of dornase alfa results in considerably increased costs as compared to standard treatment, also in critical care.

To ensure patient safety, well-powered trials with low risk of bias assessing patient- important outcome measures are needed if dornase alfa is continued to be used in adult critically ill patients outside the clinical context of CF.

## Conclusions

This systematic review with meta-analysis demonstrated that the quantity and quality of evidence for the use of dornase alfa in adult critically ill patients is very low and that there is no firm evidence for benefit or harm as compared to placebo or hypertonic saline.

## Additional files

Additional file 1:
**PRISMA flowchart.** (JPG 29 kb) Additional file 2:
**Detailed search strategy.** (DOCX 15 kb)

## Abbreviations

CF: cystic fibrosis
CI: confidence interval
DIS: diversity-adjusted required information size
*I*^2^: inconsistency factor
ICU: intensive care unit
RCT: randomised clinical trial
RR: relative risk
RRR: relative risk reduction
TSA: trial sequential analysis

## References

[1] Wagener JS, Kupfer O (2012). Dornase alfa (Pulmozyme). Curr Opin Pulm Med.

[2] Jones AP, Wallis C (2010). Dornase alfa for cystic fibrosis. Cochrane Database Syst Rev.

[3] Rahman NM, Maskell NA, West A, Teoh R, Arnold A, Mackinlay C (2011). Intrapleural use of tissue plasminogen activator and DNase in pleural infection. N Engl J Med.

[4] Dilmen U, Karagol BS, Oguz SS (2011). Nebulized hypertonic saline and recombinant human DNase in the treatment of pulmonary atelectasis in newborns. Pediatrics Int.

[5] Silverman RA, Foley F, Dalipi R, Kline M, Lesser M (2012). The use of rhDNAse in severely ill, non-intubated adult asthmatics refractory to bronchodilators: a pilot study. Respir Med.

[6] Mainz JG, Schiller I, Ritschel C, Mentzel HJ, Riethmuller J, Koitschev A (2011). Sinonasal inhalation of dornase alfa in CF: a double-blind placebo-controlled cross-over pilot trial. Auris Nasus Larynx.

[7] Thornby KA, Johnson A, Axtell S (2014). Dornase alfa for non-cystic fibrosis pediatric pulmonary atelectasis. Ann Pharmacother.

[8] Higgins JPT, Green S. Cochrane handbook for systematic reviews of interventions, version 5.1.0. 2012. http://handbook.cochrane.org/.

[9] Moher D, Liberati A, Tetzlaff J, Altman DG (2009). Preferred reporting items for systematic reviews and meta-analyses: the PRISMA statement. BMJ.

[10] Ciani O, Buyse M, Garside R, Pavey T, Stein K, Sterne JA (2013). Comparison of treatment effect sizes associated with surrogate and final patient relevant outcomes in randomised controlled trials: meta-epidemiological study. BMJ.

[11] Jammer I, Wickboldt N, Sander M, Smith A, Schultz MJ, Pelosi P (2015). Standards for definitions and use of outcome measures for clinical effectiveness research in perioperative medicine: European Perioperative Clinical Outcome (EPCO) definitions: a statement from the ESA-ESICM joint taskforce on perioperative outcome measures. Eur J Anaesthesiol.

[12] Guyatt GH, Oxman AD, Kunz R, Atkins D, Brozek J, Vist G (2011). GRADE guidelines: 2. Framing the question and deciding on important outcomes. J Clin Epidemiol.

[13] Higgins JP, Thompson SG (2002). Quantifying heterogeneity in a meta-analysis. Stat Med.

[14] Wetterslev J, Thorlund K, Brok J, Gluud C (2009). Estimating required information size by quantifying diversity in random-effects model meta-analyses. BMC Med Res Methodol.

[15] Wetterslev J, Thorlund K, Brok J, Gluud C (2008). Trial sequential analysis may establish when firm evidence is reached in cumulative meta-analysis. J Clin Epidemiol.

[16] Higgins JP, Whitehead A, Simmonds M (2011). Sequential methods for random-effects meta-analysis. Stat Med.

[17] TSA. Trial sequential analysis (TSA). The Copenhagen Trial Unit, Center for Clinical Intervention Research, Rigshospitalet. Copenhagen, Denmark. Software and manual available at www.ctu.dk/tsa; 2011.

[18] Sweeting MJ, Sutton AJ, Lambert PC (2004). What to add to nothing? Use and avoidance of continuity corrections in meta-analysis of sparse data. Stat Med.

[19] Zitter JN, Maldjian P, Brimacombe M, Fennelly KP (2013). Inhaled dornase alfa (Pulmozyme) as a noninvasive treatment of atelectasis in mechanically ventilated patients. J Crit Care.

[20] Youness HA, Mathews K, Elya MK, Kinasewitz GT, Keddissi JI (2012). Dornase alpha compared to hypertonic saline for lung atelectasis in critically ill patients. J Aerosol Med Pulm Drug Delivery.

[21] Zhang Z, Xu X, Ni H (2013). Small studies may overestimate the effect sizes in critical care meta-analyses: a meta-epidemiological study. Crit Care.

[22] Savovic J, Jones HE, Altman DG, Harris RJ, Juni P, Pildal J (2012). Influence of reported study design characteristics on intervention effect estimates from randomized, controlled trials. Ann Intern Med.

[23] Perner A, Haase N, Guttormsen AB, Tenhunen J, Klemenzson G, Aneman A (2012). Hydroxyethyl starch 130/0.42 versus Ringer’s acetate in severe sepsis. N Engl J Med.

[24] Heyland D, Muscedere J, Wischmeyer PE, Cook D, Jones G, Albert M (2013). A randomized trial of glutamine and antioxidants in critically ill patients. N Engl J Med.

[25] De Backer D, Biston P, Devriendt J, Madl C, Chochrad D, Aldecoa C (2010). Comparison of dopamine and norepinephrine in the treatment of shock. N Engl J Med.

[26] Ferguson ND, Cook DJ, Guyatt GH, Mehta S, Hand L, Austin P (2013). High-frequency oscillation in early acute respiratory distress syndrome. N Engl J Med.

[27] Ho KM, Sheridan DJ (2006). Meta-analysis of frusemide to prevent or treat acute renal failure. BMJ.

[28] Grieve R, Thompson S, Normand C, Suri R, Bush A, Wallis C (2003). A cost-effectiveness analysis of rhDNase in children with cystic fibrosis. Int J Technol Assess Health Care.

