# Supplementary figures and images for: Nebulised dornase alfa versus placebo or hypertonic saline in adult critically ill patients: a systematic review of randomised clinical trials with meta-analysis and trial sequential analysis
Source: Syst Rev. 2015 Nov 8;4:153. doi: 10.1186/s13643-015-0142-z (PMC4637143; doi:10.1186/s13643-015-0142-z)

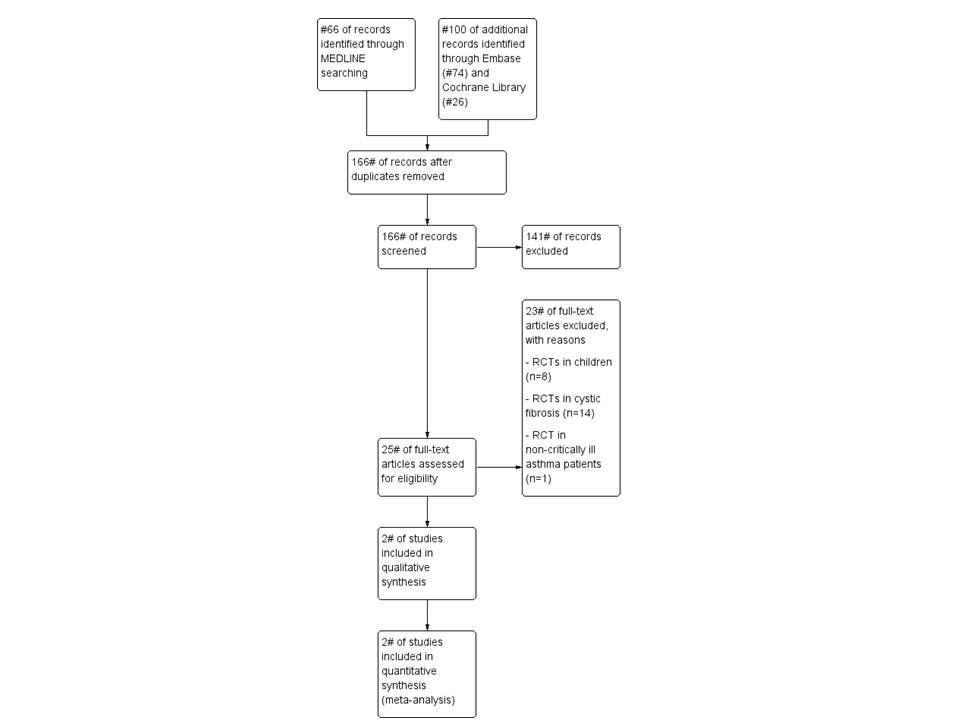

Supplement: Additional file 1: — PRISMA flowchart. (JPG 29 kb) [file 13643_2015_142_MOESM1_ESM.jpeg]
