# Supplementary material for: Nebulised dornase alfa versus placebo or hypertonic saline in adult critically ill patients: a systematic review of randomised clinical trials with meta-analysis and trial sequential analysis
Source: Syst Rev. 2015 Nov 8;4:153. doi: 10.1186/s13643-015-0142-z (PMC4637143; doi:10.1186/s13643-015-0142-z)
Supplement: Additional file 2: — Detailed search strategy. (DOCX 15 kb) [file 13643_2015_142_MOESM2_ESM.docx]

**Search strategy**

1. **MEDLINE including MeSH (January 1966 to January 2015) #66**

(((("dornase alfa"[Supplementary Concept] OR "dornase alfa"[All Fields] OR "dornase alpha"[All Fields]) OR ("dornase alfa"[Supplementary Concept] OR "dornase alfa"[All Fields] OR "recombinant human dnase"[All Fields])) OR ("dornase alfa"[Supplementary Concept] OR "dornase alfa"[All Fields] OR "rhdnase"[All Fields])) OR ("dornase alfa"[Supplementary Concept] OR "dornase alfa"[All Fields] OR "pulmozyme"[All Fields])) OR (recombinant[All Fields] AND ("humans"[MeSH Terms] OR "humans"[All Fields] OR "human"[All Fields]) AND ("deoxyribonucleases"[MeSH Terms] OR "deoxyribonucleases"[All Fields] OR "deoxyribonuclease"[All Fields])) AND Randomized Controlled Trial[ptyp]

1. **EMBASE (January 1980 to January 2015) #74**

#1 recombinant human deoxyribonuclease.mp. [mp=title, abstract, heading word, drug trade name, original title, device manufacturer, drug manufacturer, device trade name, keyword]

#2 dornase alpha.mp. [mp=title, abstract, heading word, drug trade name, original title, device manufacturer, drug manufacturer, device trade name, keyword]

#3 recombinant human DNase.mp. [mp=title, abstract, heading word, drug trade name, original title, device manufacturer, drug manufacturer, device trade name, keyword]

#4 rhDNase.mp. [mp=title, abstract, heading word, drug trade name, original title, device manufacturer, drug manufacturer, device trade name, keyword]

#5 Pulmozyme.mp. [mp=title, abstract, heading word, drug trade name, original title, device manufacturer, drug manufacturer, device trade name, keyword]

#6 1 or 2 or 3 or 4 or 5

#7 limit 6 to randomized controlled trial

1. **Cochrane Library (Issue 3, January 2015) #26**

#1 recombinant human deoxyribonuclease or dornase alpha or recombinant human DNase or rhDNase or Pulmozyme (Word variations have been searched)
